# Completed audit of the use of seclusion in the Approved Centre in Tallaght University Hospital following the introduction of an Integrated Care Pathway

**DOI:** 10.1192/bjo.2021.100

**Published:** 2021-06-18

**Authors:** Rebecca Conlan-Trant, Kate Corrigan, Peter Whitty

**Affiliations:** Department of Psychiatry, Tallaght University Hospital

## Abstract

**Aims:**

The Mental Heath Commission (MHC) is an independent body in Ireland, set up in 2002, to promote, encourage and foster high standards and good practices in the delivery of mental health services and to protect the interests of patients who are involuntarily admitted. Guidelines on the rules governing the use of seclusion are published by the MHC. These guidelines must be followed and recorded in the patient's clinical file during each seclusion episode. A Seclusion Integrated Care Pathway (ICP) was devised in 2012 for use in the Approved Centre in Tallaght University Hospital. This ICP was developed in conjunction with the MHC guidelines to assist in the recording and monitoring of each seclusion episode. Since its introduction in 2012, this ICP has become an established tool used in the Approved Centre in Tallaght University Hospital.

The aim of this audit was to assess adherence to MHC guidelines on the use of seclusion in the Approved Centre in Tallaght University Hospital 8 years after the introduction of an ICP and compare it to adherence prior to its introduction and immediately after its introduction.

**Method:**

Thirteen rules governing the use of seclusion have been published by the MHC. These include the responsibility of registered medical practitioners (RMP), nursing staff and the levels of observations and frequency of reviews that must take place during each seclusion episode. Using the seclusion register we identified a total of 50 seclusion episodes between August 2019 and July 2020. A retrospective chart review was conducted to assess documentation of each seclusion episode.

**Result:**

There was an overall improvement in adherence with MHC guidelines compared to adherence prior to the introduction of the ICP and immediately after its introduction. Areas of improvement included medical reviews, nursing reviews, informing patient of reasons for, likely duration of and circumstances that could end seclusion, and informing next of kin. The range of compliance levels across the thirteen MHC guidelines improved from 3–100% to 69–100%. Post intervention there was 100% compliance with five of the thirteen guidelines.

**Conclusion:**

The introduction of an ICP led to an overall improvement in compliance with MHC guidelines. The ICP has ensured that many of the rules governing seclusion are explicitly stated; however adjustments and revisions to the document and ongoing staff training are needed to ensure full adherence to MHC guidelines.

